# D-dimer trends elaborate the heterogeneity of risk in hospitalized patients with COVID-19: A multi-national case series from different waves

**DOI:** 10.3389/fmed.2023.1103842

**Published:** 2023-03-17

**Authors:** Diana Maria Ronderos Botero, Alaa Mabrouk Salem Omar, Martino F. Pengo, Syed Waqas Haider, Hira Latif, Gianfranco Parati, Vittorio Pengo, Alejandra Cañas Arboleda, Melissa Díaz, Claudio Villaquirán-Torres, Johanna Contreras, Sridhar Chilimuri

**Affiliations:** ^1^Department of Medicine, BronxCare Hospital Center, Bronx, New York, NY, United States; ^2^Department of Cardiology, Mount Sinai Morningside, New York, NY, United States; ^3^Department of Cardiovascular Diseases, Icahn School of Medicine at Mount Sinai, New York, NY, United States; ^4^Department of Cardiovascular, Neural and Metabolic Sciences, IRCCS Istituto Auxologico Italiano, Milan, Italy; ^5^Department of Medicine and Surgery, University of Milano-Bicocca, Milan, Italy; ^6^MedStar Heart and Vascular Institute, MedStar Washington Hospital Center, Baltimore, MD, United States; ^7^Department of Hematology and Medical Oncology, MedStar Washington Hospital Center, Baltimore, MD, United States; ^8^Department of Cardio-Thoracic-Vascular Sciences and Public Health, University of Padua, Padua, Italy; ^9^Department of Internal Medicine, Hospital Universitario San Ignacio-Pontificia Universidad Javeriana, Bogota, Colombia

**Keywords:** COVID-19, D-dimer, variability, in-hospital mortality, heterogeneity

## Abstract

**Introduction:**

Variable D-dimer trends during hospitalization reportedly result in distinct in-hospital mortality. In this multinational case series from the first and second waves, we show the universality of such D-dimer trends.

**Methods:**

We reviewed 405 patients with COVID-19 during the first wave admitted to three institutions in the United States, Italy, and Colombia, and 111 patients admitted to the U.S. site during the second wave and 55 patients during the third wave. D-dimer was serially followed during hospitalization.

**Results:**

During the first wave, 66 (15%) patients had a persistently-low pattern, 33 (8%) had early-peaking, 70 (16%) had mid-peaking, 94 (22%) had fluctuating, 30 (7%) had late-peaking, and 112 (26%) had a persistently-high pattern. During the second and third waves, similar patterns were observed. D-dimer patterns were significantly different in terms of in-hospital mortality similarly in all waves. Patterns were then classified into low-risk patterns (persistently-low and early-peaking), where no deaths were observed in both waves, high-risk patterns (mid-peaking and fluctuating), and malignant patterns (late-peaking and persistently-high). Overall, D-dimer trends were associated with an increased risk for in-hospital mortality in the first wave (overall: HR: 1.73) and stayed the same during the second (HR: 1.67, *p* < 0.001) and the third (HR: 4.4, *p* = 0.001) waves.

**Conclusion:**

D-dimer behavior during COVID-19 hospitalization yielded universal categories with distinct mortality risks that persisted throughout all studied waves of infection. Monitoring D-dimer behavior may be useful in the management of these patients.

## Introduction

Coronavirus disease 2019 (COVID-19) has been reportedly associated with a hypercoagulable state (1). An increase in fibrin degradation products (D-dimer) linked to a thrombotic state is an integral part of the COVID-19 laboratory signature (2). While clinical trials evaluating the benefit of anticoagulation are underway (3), strategies to prevent or mitigate thrombosis in these patients are currently based on limited evidence. COVID-19, however, has a wide range of symptoms and severity, and is demographically, clinically, and pathologically heterogeneous (4). One aspect of such heterogeneity can be represented by the behavior of the D-dimer levels throughout the hospitalization with COVID-19. We recently reported that the variation in D-dimer trends during the hospital course involves specific trends that resulted in distinct patterns of in-hospital mortality. Here, we report a multicenter case series from infection waves during different time points in which we show the universality of such D-dimer trends and their risk (5).

## Methods

**Figure 1:**
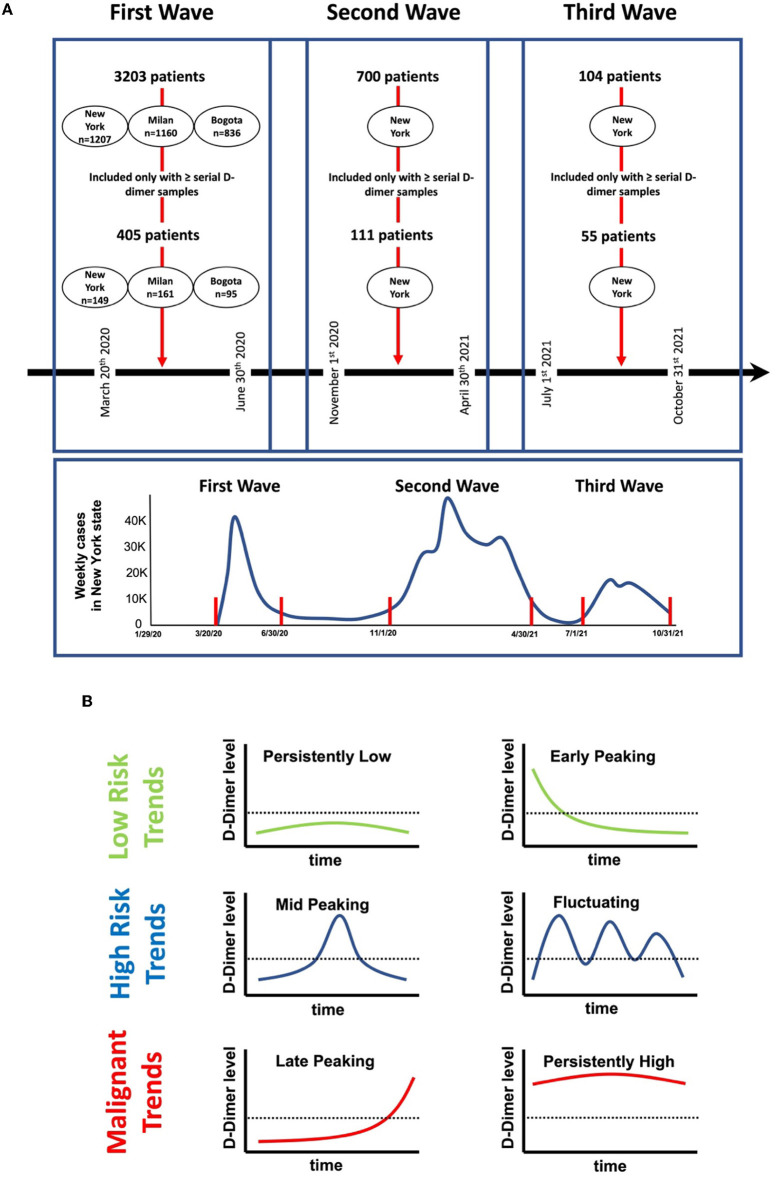
Study timelines and protocol. **(A)** Number of patients involved in the study at each center according to the defined time points in the first, second, and third waves. **(B)** Schematic representation of in-hospital D-dimer patterns observed in our study. Persistently-low: if D-dimer levels during admission were below 1,000 ng/ml and stayed below 1,000 ng/ml throughout the hospitalization. Early-peaking: D-dimer levels on admissions were >1,000 ng/ml and immediately normalized to levels <1,000 ng/ml and stayed low for the rest of the hospitalization. Mid-peaking: D-dimer levels were <1,000 ng/ml on admission, however, peaked at levels >1,000 ng/ml during the hospitalization, and then immediately decreased and stayed low for the rest of the hospitalization. Fluctuating: D-dimer levels were either low or normal during admission, however, with multiple rises and falls >1,000 ng/ml during the hospital course. Late-peaking: D-dimer levels were <1,000 ng/ml on admission and stayed low throughout the hospitalization, however, exhibited a sudden rise to levels >1,000 ng/ml at the end of the encounter. Persistently-high: D-dimer levels were >1000 ng/ml on admission which stayed high throughout the hospitalization.

In a retrospective study protocol, we defined different waves of increased infection rates during the pandemic according to the Center for Disease Control and Prevention statistics as published on their website (6). The curves reported by the CDC for the total number of weekly newly reported cases in New York State were examined. The first three peaks with stable plateaus of low reported number in-between were identified, and the onset and offset of each of these curves were identified as the time threshold for each wave. Figure 1A shows the curves for the first three waves as produced by the CDC website and the time thresholds for each wave. Accordingly, the first wave was defined as the period between 25 March 2020 and 31 June 2020. The second wave of the pandemic was defined as the period between 1 November 2020 and 30 April 2021. The third wave of the pandemic was defined as the period between 1 July 2021 and 31 October 2021. We scanned patients admitted with PCR-confirmed COVID-19 in the first wave period admitted to three different institutions representing three different continents (North America, Europe, and South America) [BronxCare Health System, New York, USA (New York site); IRCCS Istituto Auxologico Italiano, Milan, Italy (Milan Site); and Hospital San Ignacio, Bogota, Colombia (Bogota site)]. Patients who had their D-dimer followed during hospitalization (≥4 levels) until the outcome of the hospitalization (death or discharge) were included in the study. Moreover, patients admitted to the New York site in the second and third wave periods were also included in the study if they had their D-dimer followed during the hospitalization similar to those described earlier.

**Figure 2:**
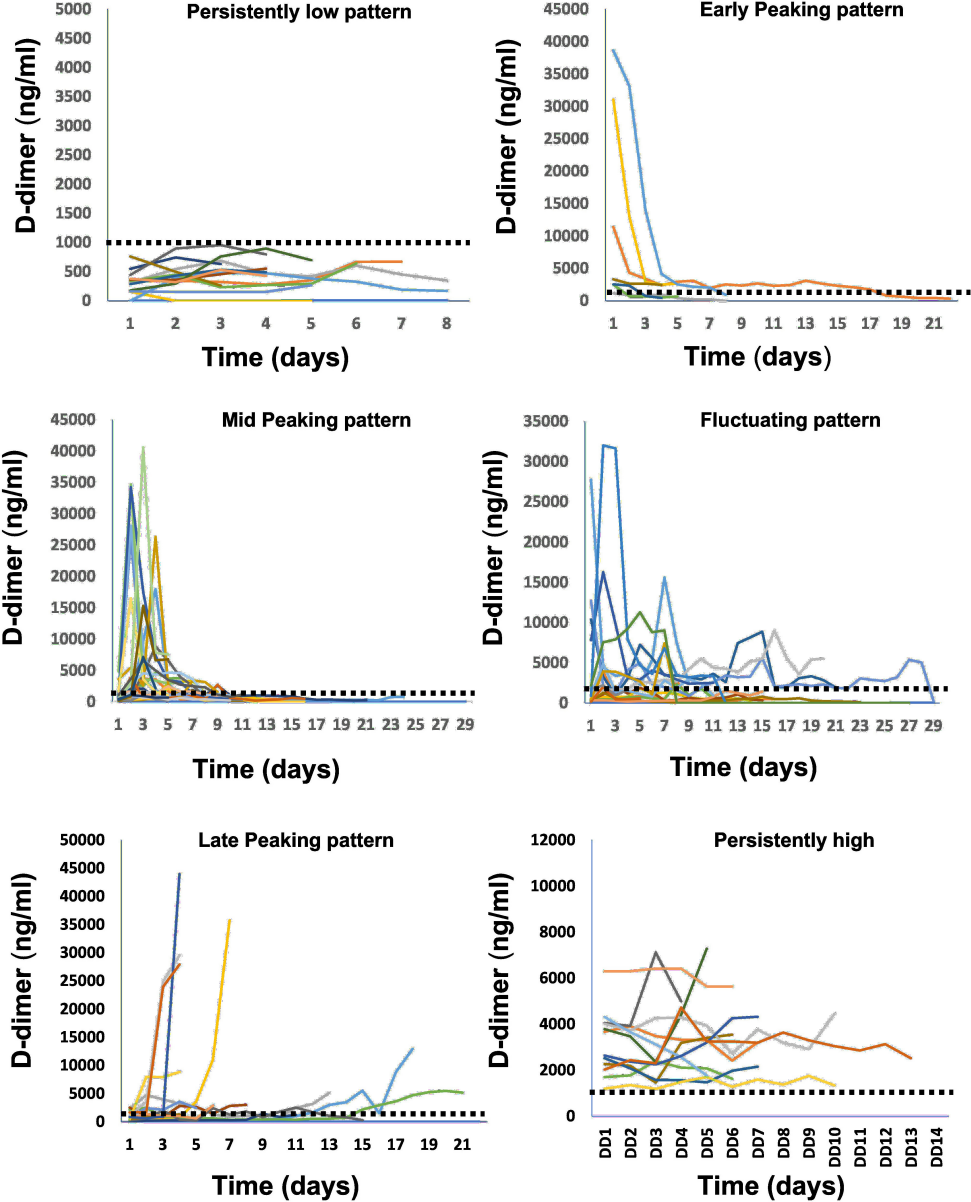
Examples of in-hospital D-dimer patterns observed in our study from different patients. Each curve represents D-dimer behavior measured from repeated samples during the hospitalization time for a separate patient.

D-dimers were classified into six different trend categories (Figure 1B) based on the behavior during hospitalization: (a) persistently-low: if D-dimer levels during admissions were ≤ 1,000 ng/ml and stayed below 1,000 ng/ml throughout the hospitalization, (b) early-peaking: D-dimer on admissions was >1,000 ng/ml and immediately or progressively normalized to levels < 1,000 ng/ml and stayed low for the rest of the hospitalization, (c) mid-peaking: D-dimer levels were < 1,000 ng/ml on admission, however, peaked to levels >1,000 ng/ml during the hospitalization, and then immediately decreased and stayed low for the rest of the hospitalization, (d) fluctuating: D-dimer levels were either low or normal during admission, however, with multiple rises and falls >1,000 ng/ml during the hospital course, (e) late-peaking: D-dimer levels that were < 1,000 ng/ml on admission and stayed low throughout the hospitalization, however, exhibited sudden rise to levels >1,000 ng/ml at the end of the encounter, and (f) persistently-high: D-dimer levels >1,000 ng/ml on admission that stayed high throughout the hospitalization. Figure 2 illustrates examples of patients' D-dimer trends.

### Statistical analyses

Categorical data are presented as numbers (%) and were compared using the chi-square test. Continuous data are presented as mean ± SD. Data were tested for normality using the Kolmogorov–Smirnov and Shapiro–Wilk tests, and accordingly, continuous data were compared using the t-test or analysis of variance (ANOVA) if they were normally distributed or the Mann–Whitney U-test if they were not normally distributed. Cox regression models and Kaplan–Meier survival curves were used to test the difference in cumulative in-hospital mortality. Differences were considered statistically significant at *p* < 0.05. All analyses will be performed with commercially available software (SPSS, version 23.0; SPSS, Inc).

## Results

During the first wave, 3,203 patients were reviewed (New York site: 1,207, Milan site: 1,160, Bogota site:836 patients) of whom 405 patients had serial D-dimer measurements and were included from the first wave from the three institutions (149 from New York site, 161 from Milan site, and 95 from Bogotá site). Moreover, 700 patients were reviewed from the New York Site during the second wave, with 111 patients having serial D-dimer measurements and being included, and 104 patients were reviewed from the New York Site during the third wave, with 55 patients having serial D-dimer measurements and being included.

Comparisons between the patients from the first, second, and third waves and the populations from the three institutions are summarized in Tables 1, 2. Briefly, patients in the second wave were more likely to be women, had less anticoagulation, more mechanical ventilation, and in-hospital deaths, while admission D-dimer was not different between the three waves. When patients from different institutes included in the first wave were compared, it was found that patients from the New York site had the highest BMI, highest mechanical ventilation, and in-hospital death. In contrast, patients from the Milan site were the oldest, most frequently male subjects, with the longest symptom onset to hospital admission and the longest hospital stay; while patients from Bogota were younger and more frequently female subjects, with the shortest symptom onset to hospital admission, the shortest hospital stay, and the most frequent anticoagulation use. It is important to note that the admission D-dimer from the three sites was not different.

**Table 1:** Comparisons between different waves.

	**First wave (*n* = 405)**	**Second wave (*n* = 111)**	**Third wave (*n* = 55)**	***p*-value**
Age, year	64.8 ± 16.3	64.1 ± 17	64.4 ± 19.5	0.681
Females, *n* (%)	192 (45)	79 (71)	22 (40)	< 0.001
BMI, *n* (%)	29.7 ± 8.4	31 ± 9.8	32 ± 8.7	0.071
Diabetes miletus, *n* (%)	130 (32)	57 (51)	26 (47)	< 0.001
Hypertension, *n* (%)	255 (63)	89 (80)	45 (82)	< 0.001
Asthma, *n* (%)	28 (7)	2 (2)	6 (11)	0.002
COPD, *n* (%)	55 (14)	1 (1)	10 (18)	0.003
Admission D-dimer, ng/ml	2,714 ± 6,646	3,962 ± 15,685	1,455 ± 2,912	0.253
Length of stay, days	16.2 ± 12.8	15.3 ± 10.6	13.4 ± 7.4	0.467
Therapeutic anticoagulation, *n* (%)	267 (62)	57 (51)	45 (82)	0.003
Mechanical ventilation, *n* (%)	124 (29)	78 (70)	20 (36)	< 0.001
In-hospital death, *n* (%)	120 (28)	67 (60)	17 (31)	< 0.001
**D-dimer trends**				0.003
Persistently-low, *n* (%)	66 (15)	22 (20)	12 (22)	
Early-peaking, *n* (%)	33 (8)	12 (11)	5 (9)	
Mid-peaking, *n* (%)	70 (16)	7 (6)	13 (24)	
Fluctuating, *n* (%)	94 (22)	24 (22)	6 (11)	
Late-peaking, *n* (%)	30 (7)	20 (18)	8 (15)	
Persistently-high, *n* (%)	112 (26)	26 (23)	11 (20)	

BMI, body mass index; COPD, chronic obstructive pulmonary disease.

**Table 2:** Comparisons between different study sites during the first wave.

**First wave**	**New York *N* = 149**	**Italy *N* = 161**	**Colombia *N* = 95**	***p*-value**
Age, year	63.7 ± 14.8	68.6 ± 15.2	59.8 ± 18.8	< 0.001
Females, *n* (%)	72 (48)	58 (36)	62 (65)	< 0.001
BMI, *n* (%)	31.3 ± 9.3	26.9 ± 4.5	26.9 ± 11	< 0.001
Symptom onset till admission, days	7 ± 6.1	11.4 ± 10	5.8 ± 4.1	< 0.001
Admission D-dimer, ng/ml	3,441 ± 9,122	2,316 ± 4,520	1,863 ± 1,743	0.168
Diabetes millitus, *n* (%)	92 (62)	26 (16)	12 (13)	< 0.001
Hypertension, *n* (%)	119 (80)	96 (60)	40 (42)	< 0.001
Asthma, *n* (%)	24 (16)	3 (2)	1 (1)	< 0.001
COPD, *n* (%)	24 (16)	18 (11)	13 (14)	0.558
Length of stay, days	16.5 ± 13	18.6 ± 13.1	11.6 ± 11	< 0.001
Therapeutic anticoagulation, *n* (%)	113 (76)	66 (41)	88 (93)	< 0.001
Mechanical ventilation, *n* (%)	81 (54)	20 (12)	23 (24)	< 0.001
In-hospital death, *n* (%)	64 (43)	41 (25)	15 (16)	< 0.001
**D-dimer trends**				< 0.001
Persistently-low, *n* (%)	22 (15)	25 (16)	19 (20)	
Early-peaking, *n* (%)	11 (7)	16 (10)	6 (6)	
Mid-peaking, *n* (%)	29 (19)	23 (14)	18 (19)	
Fluctuating, *n* (%)	21 (14)	63 (39)	10 (11)	
Late-peaking, *n* (%)	16 (11)	5 (3)	9 (9)	
Persistently-high, *n* (%)	50 (34)	29 (18)	33 (35)	

BMI, body mass index; COPD, chronic obstructive pulmonary disease.

### D-dimer trends

According to our definitions for D-dimer trends, of the 405 patients included during the first wave, 66 (15%) patients had a persistently-low pattern, 33 (8%) patients had an early-peaking pattern, 70 (16%) patients had a mid-peaking pattern, 94 (22%) patients had a fluctuating pattern, 30 (7%) patients had a late-peaking pattern, and 112 (26%) patients had a persistently-high pattern (Table 1). During the second wave, 22 (11%) patients had a persistently-low pattern, 12 (11%) patients had an early-peaking pattern, 7 (6%) patients had a mid-peaking pattern, 24 (22%) patients had a fluctuating pattern, 20 (18%) patients had a late-peaking pattern, and 26 (23%) patients had a persistently-high pattern (Table 1). During the third wave, 12 (22%) patients had a persistently-low pattern, 5 (9%) patients had an early-peaking pattern, 13 (24%) patients had a mid-peaking pattern, 6 (11%) patients had a fluctuating pattern, 8 (15%) patients had a late-peaking pattern, and 11 (20%) patients had a persistently-high pattern (Table 1).

During the first wave, patients from the Milan site had the highest number of early-peaking and fluctuating patterns, and patients from the Bogota site had the highest number of persistently-low and persistently-high patterns. Compared to the first wave, the second wave patients showed more frequent persistently-low and late-peaking D-dimer patterns, while the third wave showed more frequent late-peaking patterns (Table 2).

### Comparisons between different D-dimer patterns

Comparisons between the different D-dimer trends in all waves are shown in Table 3. In brief, there was no significant difference between the different trends regarding age, sex, BMI, or symptom onset to hospital admission. D-dimer levels on admission were significantly different between groups as can be expected from the classification. Moreover, the longest hospital stay was noted in the fluctuating and late-peaking groups, and the shortest was found for the persistently-low trend. Importantly, the lowest use of AC and mechanical ventilation were observed in the persistently-low pattern. Importantly, no in-hospital deaths were recorded in the persistently-low or the early-peaking groups, while the highest deaths occurred in the late-peaking and the persistently-high groups. Similar results were also observed in the second and third waves (Table 3).

**Table 3:** Comparisons between different trends in all waves.

	**Persistent-low**	**Early-peaking**	**Mid-peaking**	**Fluctuating**	**Late-peaking**	**Persistent-high**	***p*-value**
**1st wave**							
Number	66	30	70	94	30	112	
Age, years	62.3 ± 15.8	65.8 ± 15.8	60.7 ± 16.6	66.8 ± 17.4	65.1 ± 18.9	66.7 ± 14.9	0.116
Females, *n* (%)	32 (48)	19 (63)	39 (56)	56 (60)	13 (43)	60 (54)	0.711
BMI, kg/m^2^	31 ± 11.8	26.7 ± 4	29.7 ± 7.1	28.8 ± 7	31.4 ± 6	30.3 ± 9.9	0.444
Symptoms onset till admission, days	8.6 ± 10.5	7.6 ± 6	8.1 ± 6.6	9.8 ± 9.3	9.6 ± 10.7	6.5 ± 4.9	0.178
Admission D-dimer, ng/ml	926.2 ± 1,387	2787.8 ± 6,825	873 ± 838	2,031 ± 4,169	1,110 ± 1,143	6,388 ± 11,155	< 0.001
Length of stay, days	12.7 ± 10	15.5 ± 13	15.9 ± 13	24.2 ± 13.8	17.8 ± 13.2	13.6 ± 11.6	< 0.001
Therapeutic anticoagulation, *n* (%)	24 (36)	22 (73)	52 (74)	59 (63)	22 (73)	82 (73)	< 0.001
Mechanical ventilation	8 (12)	1 (3)	22 (31)	32 (34)	14 (47)	39 (35)	< 0.001
In-hospital death, *n* (%)	0 (0)	0 (0)	13 (19)	32 (34)	14 (47)	51 (46)	< 0.001
**2**^n*d*^ **wave**							
Number	22	12	7	24	20	26	
Age, years	57.6 ± 15.4	65.7 ± 13	69 ± 11.5	67.4 ± 15	65 ± 19	68.3 ± 15	0.215
Females, *n* (%)	10 (45)	6 (50)	3 (43)	14 (58)	11 (55)	18 (69)	0.614
BMI, kg/m^2^	28.8 ± 8.7	30.5 ± 6.6	31.9 ± 7	31.6 ± 5.9	33.8 ± 12.4	33.9 ± 10.7	0.441
Admission D-dimer, ng/ml	390 ± 247	4,080 ± 4,455	468 ± 242	4,121 ± 10,782	404 ± 182	13,033 ± 32,596	< 0.001
Length of stay, days	13.5 ± 8.6	16.4 ± 5.9	16.8 ± 3.6	23.2 ± 12.5	13.4 ± 6.5	17.8 ± 13.2	0.014
Therapeutic anticoagulation, *n* (%)	3 (14)	7 (58)	6 (86)	16 (67)	7 (35)	11 (42)	0.001
Mechanical ventilation	7 (32)	4 (33)	1 (14)	18 (75)	16 (80)	22 (85)	< 0.001
In-hospital death, *n* (%)	0 (0)	0 (0)	1 (14)	17 (71)	17 (85)	24 (92)	< 0.001
**3**^rd^ **wave**							
Number	12	5	13	6	8	11	
Age, years	69.2 ± 19.6	74 ± 11.7	60.3 ± 15	53 ± 26.6	69.5 ± 13.9	61.7 ± 14.1	0.248
Females, *n* (%)	7 (58)	2 (40)	3 (23)	1 (17)	5 (63)	3 (27)	0.216
BMI, kg/m^2^	32 ± 8.1	25 ± 3.4	29.3 ± 4.9	33.8 ± 11	38.3 ± 10	31.4 ± 9.1	0.09
Admission D-dimer, *n*g/ml	339 ± 109	2,002 ± 882	426 ± 232	516 ± 231	398 ± 179	5,025 ± 5,255	< 0.001
Length of stay, days	16.5 ± 14.9	23.2 ± 19.1	18.6 ± 14.7	12.1 ± 8.6	13.9 ± 5.1	13.4 ± 6.7	0.612
Therapeutic anticoagulation, *n* (%)	5 (42)	4 (80)	12 (92)	5 (83)	7 (88)	11 (100)	0.009
Mechanical ventilation	0 (0)	0 (0)	4 (31)	5 (83)	5 (63)	5 (45)	0.002
In-hospital death, n (%)	0 (0)	0 (0)	3 (23)	1 (17)	6 (75)	6 (55)	0.002

**Figure 3:**
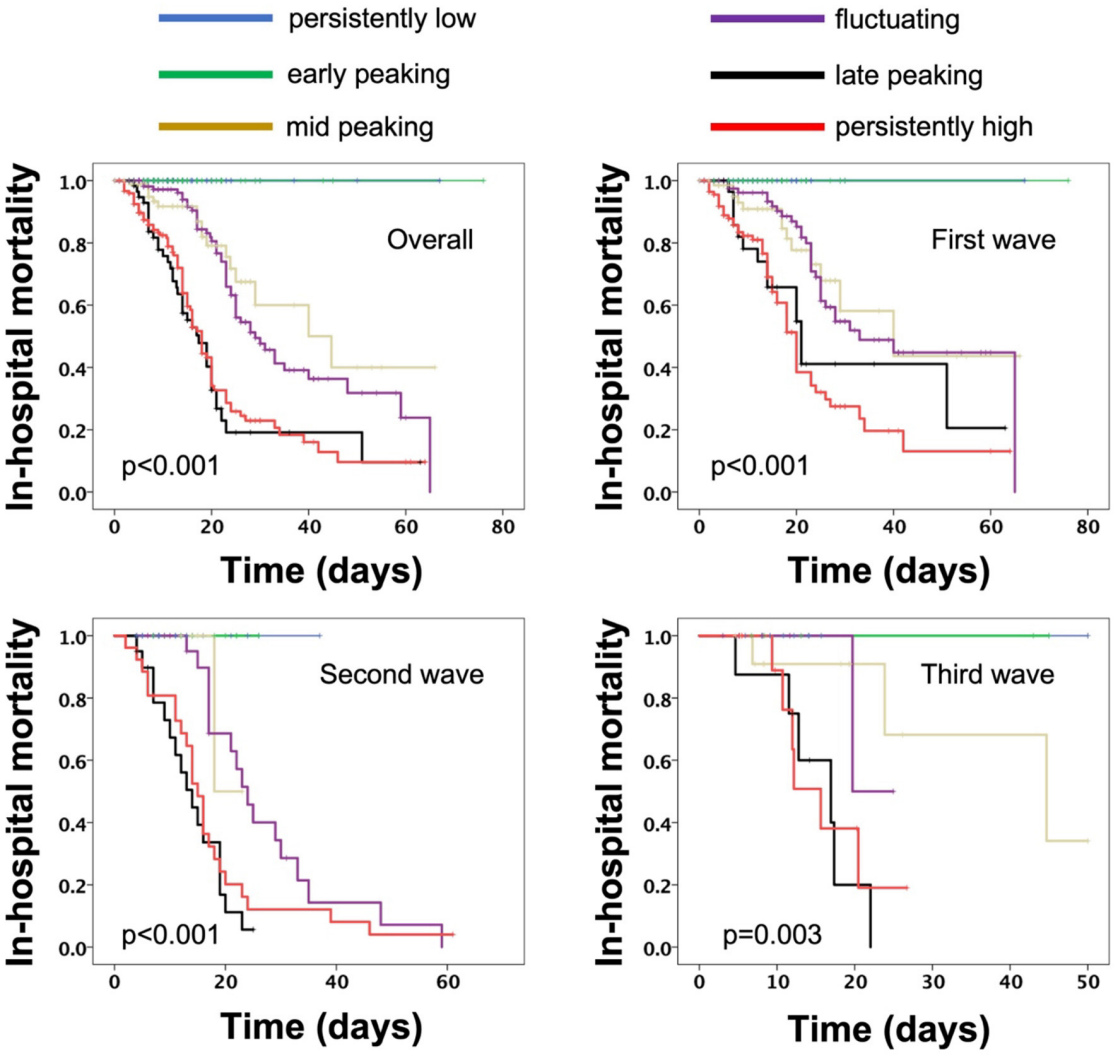
Kaplan–Meier curves for in-hospital mortality overall as well as during the first, second, and third waves.

Kaplan–Meier curves revealed that different patterns of D-dimer were highly significantly different in terms of in-hospital mortality (Figure 3). Importantly, the patterns of risk observed were similar in all waves. Based on the curves, we found that the patterns can be classified according to in-hospital mortality risk into low-risk patterns (persistently-low and early-peaking), where no deaths were observed in all waves, high-risk patterns (mid-peaking and fluctuating), and malignant patterns (late-peaking and persistently high).

Cox-regression analysis revealed that, overall, D-dimer trends are associated with an increased risk for in-hospital mortality in the first wave (overall: HR: 1,73, *p* < 0.001; New York site: RR: 1.58, *p* < 0.001; Milan site: RR: 1.82, *p* < 0.001; Bogota site: 1.9, *p* = 0.008) and stayed the same during the second wave (HR: 1.67, *p* < 0.001) and the third wave (HR: 2, *p* = 0.002).

**Figure 4:**
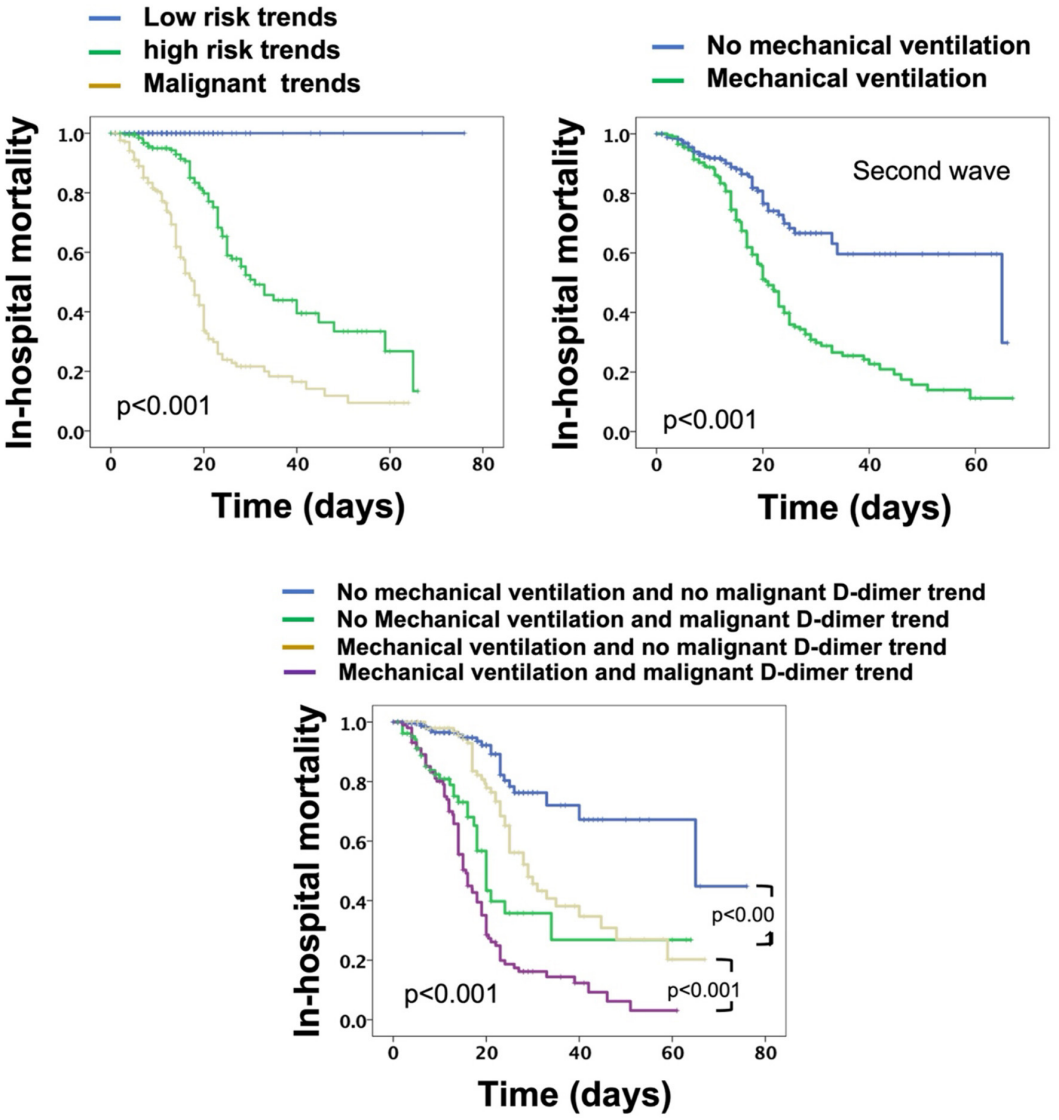
Kaplan–Meier curves for overall in-hospital mortality for patients stratified based on D-dimer trends, mechanical ventilation, and both.

Compared to low and high risk (Figure 4), the malignant risk patterns were associated with a significant RR of in-hospital mortality in the first wave (RR:3.64, *p* < 0.001, New York site: RR: 2.87 *p* < 0.001; Milan site: RR: 3.85, *p* < 0.001; Bogota site: 7.4, *p* = 0.009) as well as the second wave (RR: 3.83, *p* < 0.001), and the third wave (RR: 9.5, *p* = 0.001).

Univariate Cox-regression models were initiated for predictors of in-hospital mortality in all patients from all sites and across all waves (Table 4). It was found that age, hypertension, diabetes, mechanical ventilation, and D-dimer trends were all associated with increased risk for in-hospital mortality. Multivariate regression showed that only D-dimer trends and mechanical ventilation were associated with increased risk for mortality; however, D-dimer trends were a stronger predictor compared to mechanical ventilation (Table 4). When patients were stratified based on mechanical ventilation and malignant D-dimer trends, it was found that patients with malignant D-dimer trends were associated with a higher risk of in-hospital mortality both in those who were mechanically ventilated and those who did not require mechanical ventilation (Figure 4).

**Table 4:** Overall predictors of in-hospital mortality.

	**Univariate**			**Multivariate**		
	**HR**	***p*** **-value**	**95%CI**	**HR**	***p*** **-value**	**95%CI**
Age, years	1.01	0.015	1.01–1.02	1.01	0.178	0.99–1.02
Females, *n* (%)	0.79	0.117	0.59–1.06	-	-	-
Diabetes mellitus, *n* (%)	1.43	0.012	1.09–1.95	1.21	0.216	0.89–1.6
Hypertension, *n* (%)	1.6	0.007	1.14–2.24	1.36	0.101	0.94-2
Asthma, *n* (%)	0.8	0.481	0.42–1.5	-	-	-
COPD, *n* (%)	0.82	0.454	0.49–1.38	-	-	-
Admission D-dimer, ng/ml	1.01	0.09	0.99–1.02	-	-	-
Therapeutic anticoagulation, *n* (%)	0.96	0.681	0.68–1.29	-	-	-
Mechanical ventilation	2.6	< 0.001	1.85–3.56	1.9	< 0.001	1.4–2.7
**DD trends**						
Overall	3.8	< 0.001	2.9–5	3.4	< 0.001	2.6–4.6
High risk	4.2	< 0.001	3.1–5.7	3.6	< 0.001	2.6–5

## Discussion

In this case series, we report D-dimer patterns during hospitalization in patients with COVID-19 that show distinct mortality behavior. Six different patterns were observed (persistently-low, early-peaking, mid-peaking, fluctuating, late-peaking, and persistently-high). While we noted a progressively increasing risk of in-hospital death in these patterns, we also noted that the persistently-low and early-peaking are benign patterns associated with no mortality in our report. Mid-peaking and fluctuating patterns, in contrast, are patterns associated with elevated risk for in-hospital mortality, and late-peaking and persistently-high D-dimer were malignant patterns associated with the highest in-hospital mortality. Patients with the malignant D-dimer trends were noted to have an elevated risk of in-hospital mortality after adjustment for co-variates and regardless of the requirement of mechanical ventilation. Importantly, these patterns and their associated risk seem to be universal among patients from different institutes with expected different genetic and ethnic backgrounds, and similar patterns were also observed in different waves of the pandemic.

### D-dimer behavior as an example of COVID-19 heterogeneity

Our observation confirms the clinical and pathological heterogeneity in patients with COVID-19 (4, 7) and provides an example of such heterogeneity through in-hospital D-dimer behavior. The laboratory signature of hospitalized patients with COVID-19 indicated that increased D-dimer levels are an integral part of the disease that is associated with worse outcomes and may be linked to a thrombotic state (9). Several studies have suggested the predictive ability of D-dimer in COVID-19 for worse outcomes; however, such studies focused on point measurement of D-dimer, especially during admission (10, 13). Our observation here suggests that elevated D-dimer on admission is not a pre-requisite for poor outcomes in patients with COVID-19. In fact, elevated D-dimer during admission may be associated with benign outcomes if the D-dimer decreases and stays low, while low D-dimer on admission may be associated with worse outcomes if D-dimer elevates once or more during the hospital stay. As such, our observation suggests that worse outcomes of COVID-19 are associated with specific patterns of D-dimer behavior during hospitalization rather than point-time measured values. The patterns observed suggest that worse outcomes are linked to a “later elevation” of D-dimer (during the hospitalization or toward the end of the encounter) or “delayed normalization” of D-dimer, and, vice versa, better outcomes are linked to earlier and continuous normalization of D-dimer.

Clinically, these findings seem of interest at least to guide the decisions in hospitalized patients with COVID-19. The role of D-dimer in the course of management of COVID-19 in all stages (pre-hospitalization, during hospitalization, and after discharge) is expanding, and its use to guide medical therapeutics such as anticoagulation is progressing despite early suspicion (8). In one prior study, it was found that the rate and the magnitude of the rise in D-dimer within the first 10 days in hospitalized patients with COVID-19 are associated with poor outcomes. In that study, this D-dimer behavior was found to be associated with venous thromboembolism but not mortality (12).

Moreover, D-dimer levels during hospitalization have been recently reported to be associated with the risk of worse outcomes in patients with COVID-19 (11). In a recent study, the patterns of D-dimer during hospitalization were associated with higher risk than static measurements (11) indicating that an increasing D-dimer trend during hospitalization is associated with worse risk compared to stable or decreasing D-dimer levels. It is to be noted, however, that a clear differentiation of a normal cutoff value was not identified, and the inclusion criteria involved ≥3 D-dimer levels within 21 days of hospital admission which may have led to significant variation in D-dimer levels that can pass undetected between measured samples. Comparatively, in our report, patients were classified based on the lowest cutoff value reported in previous studies (1,000 ng/ml). The in-hospital D-dimer trends noted in our study somewhat differed in patterns and significance. First, because of the more frequent sampling in our study, more changes could be captured allowing for the identification and differentiation of the increasing D-dimer during hospitalization into three different groups (fluctuating D-dimer, mid-peaking, and late-peaking) compared to “increasing levels” in the aforementioned study. Second, in our study, we differentiated stable patterns into persistently-low and persistently-high. Third, the decreasing pattern in our study was a low-risk pattern compared to a higher risk for the same group in the aforementioned study and that can be explained by the immediate normalization of D-dimer in our report. Finally, the group of patients with “persistently increased” D-dimer was the sickest group of patients and was associated with the worst risk of outcomes. While it is unclear whether differences between both studies are reproducible, similarities in patterns do exist, pointing toward a level of heterogeneity among patients with COVID-19 previously underappreciated.

We acknowledge the limitation of the observational nature of our case series report with small sample size, and conclusions should not be drawn until our findings are confirmed in large randomized clinical trials. Moreover, the effect of vaccination on the noted D-dimer trends was not conducted, and the expected taming effect of vaccination on the trends cannot be seen in the current report. It should also be emphasized that D-dimer behaviors noted in our study do not seem to be the governing factor behind the disease's extensive heterogeneity, as a large number of co-variates are suspected. D-dimer behavior is rather just a representation of how stratifying patients in such a manner may uncover previously under-detected effects such as the stratification done for the mechanical ventilation done in our study. While studying the nature and explanation of such heterogeneity is beyond the scope of the current report, it seems that such heterogeneity involves all demographic, clinical, and laboratory aspects of the disease. Accordingly, more in-depth large systematic prospective studies and retrospective meta-analyses taking into consideration the reported finding of D-dimer behavior in addition to other factors contributing to heterogeneity are needed to support our hypothesis. Finally, it is unknown whether the current observations are specific to patients with COVID-19, and further studies should compare D-dimer levels followed in patients between COVID-19 and other causes of elevated D-dimer in hospitalized patients.

## Conclusion

Coronavirus disease 2019 is a thrombo-inflammatory disease that is both dynamic and heterogeneous. D-dimer behavior during hospitalization is an important example of such heterogeneity and yielded categories with a distinct risk of in-hospital mortality. Such patterns seem to be universal between different hospitals from different geographic locations despite the use of different anticoagulation approaches and occurred in similar fashions in all pandemic waves. Monitoring D-dimer behavioral categories may be useful in the management of these patients regardless of the need for mechanical ventilation. Further studies are needed to determine whether D-dimer category-guided management improves outcomes in patients with COVID-19.

## Data availability statement

The raw data supporting the conclusions of this article will be made available by the authors, upon reasonable written request.

## Ethics statement

The studies involving human participants were reviewed and approved by BronxCare Hospital Center IRB. Written informed consent for participation was not required for this study in accordance with the national legislation and the institutional requirements.

## Author contributions

DB, AO, and MP: conceptualization, hypothesis generation, data collection, statistical analysis, manuscript preparation, and revision. SH, HL, GP, VP, AA, MD, CV, JC, and SC: statistical analysis, manuscript preparation, and supervision. All authors contributed to the article and approved the submitted version.
