# Corrigendum: D-dimer trends elaborate the heterogeneity of risk in hospitalized patients with COVID-19: a multi-national case series from different waves

**DOI:** 10.3389/fmed.2023.1205719

**Published:** 2023-05-10

**Authors:** Diana Maria Ronderos Botero, Alaa Mabrouk Salem Omar, Martino F. Pengo, Syed Waqas Haider, Hira Latif, Gianfranco Parati, Vittorio Pengo, Alejandra Cañas Arboleda, Melissa Díaz, Claudio Villaquirán-Torres, Johanna Contreras, Sridhar Chilimuri

**Affiliations:** ^1^Department of Medicine, BronxCare Hospital Center, Bronx, New York, NY, United States; ^2^Department of Cardiology, Mount Sinai Morningside, New York, NY, United States; ^3^Department of Cardiovascular Diseases, Icahn School of Medicine at Mount Sinai, New York, NY, United States; ^4^Department of Cardiovascular, Neural and Metabolic Sciences, IRCCS Istituto Auxologico Italiano, Milan, Italy; ^5^Department of Medicine and Surgery, University of Milano-Bicocca, Milan, Italy; ^6^MedStar Heart and Vascular Institute, MedStar Washington Hospital Center, Baltimore, MD, United States; ^7^Department of Hematology and Medical Oncology, MedStar Washington Hospital Center, Baltimore, MD, United States; ^8^Department of Cardio-Thoracic-Vascular Sciences and Public Health, University of Padua, Padua, Italy; ^9^Department of Internal Medicine, Hospital Universitario San Ignacio-Pontificia Universidad Javeriana, Bogota, Colombia

**Keywords:** COVID-19, D-dimer, variability, in-hospital mortality, heterogeneity

In the published article, there was an error regarding the affiliations for Gianfranco Parati and Martino F. Pengo. They both have the following affiliations:

Department of Medicine and Surgery, University of Milano-Bicocca, Milan, Italy and Department of Cardiovascular, Neural and Metabolic Sciences, IRCCS Istituto Auxologico Italiano, Milan, Italy.

The authors apologize for this error and state that this does not change the scientific conclusions of the article in any way. The original article has been updated.

